# Genomic changes underpinning the emergence of a successful *Mycobacterium tuberculosis* Latin American and Mediterranean clonal complex

**DOI:** 10.3389/fmicb.2023.1159994

**Published:** 2023-06-22

**Authors:** Naira Dekhil, Helmi Mardassi

**Affiliations:** Unit of Typing and Genetics of Mycobacteria, Laboratory of Molecular Microbiology, Vaccinology, and Biotechnology Development, Institut Pasteur, Tunis, University of Tunis El Manar, Tunis, Tunisia

**Keywords:** positive selection, *Mycobacerium tuberculosis*, L4.3/LAM, ESX/type VII secretion systems, whole genome sequencing, clonal complex, successful evolution

## Abstract

**Introduction:**

The Latin American and Mediterranean sublineage (L4.3/LAM) is the most common generalist sublineage of *Mycobacterium tuberculosis* lineage 4 (L4), yet certain L4.3/LAM genotypes appear to be confined to particular geographic regions. This is typically the case of a L4.3/LAM clonal complex (CC), TUN4.3_CC1, which is the most preponderant in Tunisia (61.5% of L4.3/LAM).

**Methods:**

Here, we used whole-genome sequencing data of 346 globally distributed L4 clinical strains, including 278 L4.3/LAM isolates, to reconstruct the evolutionary history of TUN4.3_CC1 and delineate critical genomic changes underpinning its success.

**Results and Discussion:**

Phylogenomic coupled to phylogeographic analyses indicated that TUN4.3_CC1 has evolved locally, being confined mainly to North Africa. Maximum likelihood analyses using the site and branch-site models of the PAML package disclosed strong evidence of positive selection in the gene category “cell wall and cell processes” of TUN4.3_CC1. Collectively, the data indicate that TUN4.3_CC1 has inherited several mutations, which could have potentially contributed to its evolutionary success. Of particular interest are amino acid replacements at the *esxK* and *eccC2* genes of the ESX/Type VII secretion system, which were found to be specific to TUN4.3_CC1, being common to almost all isolates. Because of its homoplastic nature, the *esxK* mutation could potentially have endowed TUN4.3_CC1 with a selective advantage. Moreover, we noticed the occurrence of additional, previously described homoplasic nonsense mutations in *ponA1* and Rv0197. The mutation in the latter gene, a putative oxido-reductase, has previously been shown to be correlated with enhanced transmissibility *in vivo*. In sum, our findings unveiled several features underpinning the success of a locally evolved L4.3/LAM clonal complex, lending further support to the critical role of genes encoded by the ESX/type VII secretion system.

## Introduction

*Mycobacterium tuberculosis*, the main causative agent of human tuberculosis (TB), is known as the “White Plague” (Dubos and Dubos, 1953; Daniel, 2006). Indeed, notwithstanding the tremendous global effort to contain this scourge and the availability of potent drugs and a vaccine, TB remains the top infectious killer worldwide with an estimated 10.6 million cases and 1.6 million deaths annually (World Health Organization, 2022). The recent advent of COVD-19, along with the impact of HIV co-infection and drug resistance, have made TB elimination a hard-to-reach goal (Abdool Karim and Abdool Karim, 2020).

Aside from *M. tuberculosis sensu stricto*, other closely related bacterial species can also cause TB, all of which are referred to as the *Mycobacterium tuberculosis* complex (MTBC) (Brosch et al., 2002; Brites and Gagneux, 2017). The latter complex encompasses nine human-adapted phylogenetic lineages (L1 to L9) (Ngabonziza et al., 2020; Coscolla et al., 2021; Silva et al., 2022) and four lineages adapted to various wild and domestic animal species (Brites et al., 2018). The advent of whole genome sequencing (WGS) has made it clear that the global success of TB is mainly driven by L2 and L4. While L2 (mainly comprised of the W-Beijing family of strains) is particularly predominant in Asia, but also present in other parts of the world, L4 (also known as the Euro-American lineage) appears to be the most globally widespread, being prevalent in Asia, Europe, Africa and America (Stucki et al., 2016; O’Neill et al., 2019). Depending on the breadth of their ecological niche, L4 could be split into generalists (L4.1.2/Haarlem, L4.3/LAM and L4.10/PGG3) and specialists (L4.1.3/Ghana, L4.5, L4.6.1/Uganda and L4.6.2/Cameroon) sublineages (Stucki et al., 2016).

Overall, the structured phylogeographical picture of these lineages reflects longstanding host-pathogen associations (Gagneux, 2012; Comas et al., 2013; Brites and Gagneux, 2015; Correa-Macedo et al., 2019).

Because *M. tuberculosis* L4 is the most prevalent lineage worldwide, it has recently been the focus of increasing interest. It is now well established that dispersal of this lineage has been largely shaped by colonial migration out of Europe, with repeated sourcing from this continent concomitant with European colonizing events, 1,600–1900 CE (Stucki et al., 2016; Brynildsrud et al., 2018; O’Neill et al., 2019). Ten L4 sublineages (L4.1.1 to L4.10) have been identified, with the L4.3/LAM (Latin American & Mediterranean) sublineage being the most frequently encountered globally, particularly in the Euro Mediterranean region and Latin America (Mokrousov et al., 2005; Stucki et al., 2016). The global population of L4.3/LAM encompasses three major phylogenetic branches that could be distinguished by large deletions RD115 and RD174, and by spoligotype SIT33. L4.3/LAM likely originated in the Western Mediterranean region, giving rise to its earliest RD115 branch, followed by RD174 and SIT33 spoligotype. Europe, and most likely North Africa, represent a major early step in the evolutionary history of L4.3/LAM, from where it radiated all over the world (Mokrousov et al., 2016; Stucki et al., 2016; Brynildsrud et al., 2018; O’Neill et al., 2019). The relative success of this notorious generalist sublineage may reflect its increased ability to adapt to new host populations (Stucki et al., 2016; Brynildsrud et al., 2018).

We have shown previously that the success of L4.3/LAM in Tunisia stems mainly from the expansion of a single clonal complex (CC), termed TUN4.3_CC1 (Skhairia et al., 2021), which represents 61.5% of the L4.3/LAM population. Unlike the much less prevalent TUN4.3_CC2 (14.68%), TUN4.3_CC1 lacks RD115 deletion, the earliest deletion that occurred in the L4.3/LAM sublineage, suggesting its relative ancient origin. In addition, TUN4.3_CC1 displays higher mean allelic richness and predominates throughout the entire sampled region, indicating that it is long-established in the host population. Furthermore, maximum likelihood analysis revealed that while TUN4.3_CC1 has been undergoing a demographic expansion since 131 years ago, TUN4.3_CC2 population, by contrast, was found under contraction (Skhairia et al., 2023). These findings have prompted us to undertake an in-depth analysis, based on WGS data, aimed at deciphering the origin(s) of the L4.3/LAM population in Tunisia and delineating the evolutionary forces that could have contributed to the particular success of TUN4.3_CC1.

### Materials and methods

### Global L4.3/LAM genome datasets

To investigate the phylogenetic placement of Tunisian L4.3/LAM clinical strains in a worldwide context, we used a globally distributed genome dataset consisting of 278 L4.3/LAM isolates, available at the European Nucleotide Archive (ENA), covering a wide temporal and geographic range (Supplementary Table S1). This dataset includes 41 genomes consisting of TUN4.3_CC1 (*n* = 17), TUN4.3_CC2 (*n* = 11), and 3 singletons (SING) that we have previously reported (Skhairia et al., 2021), and ten published genomes (Bouzouita et al., 2019), nine of which were found to belong to the major TUN4.3_CC1, and one genome being related to TUN4.3_CC2. Genomic data were analyzed using an in-house automated pipeline including open source softwares. Briefly, the quality control of raw sequencing data was done using FASTQC (Andrews, 2010). Both adapters and low-quality bases with a Phred quality score less than 20 were clipped using Trimmomatic (Bolger et al., 2014) and only reads with minimum length of 36 bases long were used in the downstream analysis. Three different aligners, Burrows-Wheeler Alignment Tool (BWA) (Li and Durbin, 2009), Novoalign^1^ and SMALT (Ponstingl and Ning, 2010), were used to map PE reads to the genome sequence of *M. tuberculosis* H37Rv reference strain (Genbank: AL123456.3). Local realignment and de-duplication of alignment files were performed using the Genome Analysis Toolkit (GATK) (McKenna et al., 2010) and Picard (version 1.107). VCF files including single nucleotide polymorphisms (SNPs) and Insertions/Deletions (In/Dels) were generated from each alignment file using GATK. Variants were then annotated referring to Tuberculist server (Lew et al., 2011). SNPs located in repetitive sequences such as PE/PPE genes and mobile elements, were excluded from the analysis. Only SNPs that were sequenced at a depth of at least 10 and present in at least 70% of the reads were included in this study. SpolPred software (Coll et al., 2012) was used to predict spoligotypes, *in silico*. Tunisian Raw data used in this study were deposited in the European Nucleotide Archive (accession no. PRJEB39509). Drug resistance-conferring mutations and lineage-specific SNPs were excluded from this analysis.

### Maximum likelihood phylogenetic inference

Polymorphic positions in all strains were annotated and extracted using snpToolkit (Namouchi, 2019) and a total of 15,575 variable sites were selected for phylogenetic and genetic analysis. A maximum-likelihood (ML) phylogeny was reconstructed with all lineage 4.3 genomes in Mega5 (Hall, 2013) using a general time reversible (GTR) model with four rate classes, and 100 bootstrap replicates were performed to assess statistical support. Trees were visualized and edited in Figtree v1.4.4 (Rambaut, 2014).

Pairwise SNP distances were calculated with the *ape*-package and the *dna.dist* function in R version 3.6.3 (Paradis et al., 2004). For the comparison of pairwise number of SNPs between L4.3/LAM strains, SNP distance between each isolate of a given country and each Tunisian strain was calculated, and a distribution of the pairwise distance plotted. Wilcoxon rank sum and Kruskal-Wallis tests were used to test for differences between continents as data did not show a normal distribution.

### Biogeographical analyses

The biogeographic histories of L4.3/LAM CCs were inferred using Statistical-Dispersal Analysis (S-DIVA) and Bayesian Binary MCMC (BBM) implemented in RASP v4.0 (Yu et al., 2020). For this purpose, the following distribution ranges were considered: North Africa, Africa, Europe, Americas, and Asia.

### Searching for imprints of positive selection

Evidence of positive selection in a protein’s amino acid sequence is generally assessed by comparing the nonsynonymous rate (dN) with the synonymous rate (dS). When the rate ratio *ω* = dN/dS > 1, the nonsynonymous rate is greater than the synonymous rate and this is interpreted as evidence for the action of positive selection. Purifying selection is inferred when *ω* < 1, whereas neutral evolution is assumed when *ω* = 1.

Genes carrying SNPs were grouped according to the different classes of gene categories based on the classification of Tuberculist (Lew et al., 2011). For each gene category, a single file was created containing the concatenated sequences of all genes in that category. These files were used as input, for a codon-by-codon analysis using CODEML as implemented in the software package PAML (Phylogenetic Analysis by Maximum Likelihood) (version 4.10.6) (Yang, 2007). We used *site* models where codon sites are allowed to fall into categories depending on their *ω* values. Five site models (M0, M1a, M2a, M7, and M8) were tested to detect signatures of positive selection across the genome sequence of the successful TUN4.3_CC1 clonal complex. First, we compared a “nearly neutral model,” M1a, to a “positive selection” model, M2a. The model M1a allows 2 categories of codon sites in *p* 0, and *p* 1 proportions, with *ω* 0 < 1 and, *ω* 1 = 1, whereas M2a adds an additional category of codons (*p* 2), with *ω* 2 that is free to vary above 1, thus modeling positive selection. Next, we compared *site* models M7 and M8. M7 specifies a neutral model similar to M1a, but the sites affected by negative selection approximate a beta distribution with parameters (*p* and *q*) estimated from the data. M7 is compared to M8 (selection) which allows an extra category of positively selected sites.

*Branch-site* models were also used to detect positive selection that affects only a few sites on specific lineages (Álvarez-Carretero et al., 2023). TUN4.3_CC1 branches under test for positive selection are the foreground branches, whereas all other L4 sublineage branches on the tree are the background branches. Extended data containing all scripts and codeml ctl files are found in https://github.com/Naira-ipt/M.-tuberculosis-Gene-Categories-PAML.

For the background branches, there are two classes of sites, the conserved sites with 0 < *ω*0 < 1 and the neutral sites with *ω*1 = 1. For the foreground branches, some of those sites become under positive selection with *ω*2 > 1. Positive selection or the presence of sites with *ω*2 > 1 is tested by comparing this model (M2a) with a null model in which *ω*2 = 1 is fixed (M2b), using a 50:50 mixture of 0 and χ21 as the null distribution (Álvarez-Carretero et al., 2023).

The comparison between models was assessed using Likelihood-Ratio Tests (LRTs). A significantly higher likelihood of the alternative model than that of the null model indicates positive selection in the data set examined. For models comparisons, we used degree of freedom, df = 2 for M1 vs. M2, M7 vs. M8 and df = 1 for M2a vs. M2b. For each analysis, correction for multiple testing (Bonferroni correction) was applied. Only in cases where LRT proved significant (*value of p* of Chi square < 0.05), was the Bayes empirical Bayes (BEB) procedure used to calculate the posterior probabilities (PPs) to identify sites under positive selection.

To search for homoplastic sites, we used HomoplasyFinder, and sites with a consistency index less than 1 were generally considered as homoplasies (Crispell et al., 2019).

Furthermore, mutations in each of the two main CCs that might impact on the function(s) of the encoded product were predicted by SIFT (Sorting Intolerant From Tolerant) implemented in the software SIFT4G (v2.1) (Ng and Henikoff, 2003). Here we considered only those mutations that occurred in 80% or more (evolving toward fixation) of the L4.3 sublineage CC populations.

### Distribution of homoplastic A58T nsSNPs in the *esxK* gene across *Mycobacterium tuberculosis* lineages and geographic regions

To map A58T *esxK* nsSNP in *M. tuberculosis* genomes from different lineages (L1 to L7), we selected 458 clinical strains from the five continents, including the 41 Tunisian L4.3 isolates (Supplementary Table S7). A maximum likelihood was constructed using IQ-TREE (version 1.6) (Nguyen et al., 2015).

## Results

### Placement of Tunisian strains within a global L4.3/LAM WGS-based phylogeny

To explore the evolutionary history of L4.3/LAM strains prevailing in Tunisia, we performed phylogenetic reconstructions using a global L4.3/LAM genome dataset consisting of 278 isolates originating from 52 countries, including 41 L4.3/LAM WGS data from Tunisia. Overall, 15,575 SNPs were used to infer the phylogenetic placement of Tunisian L4.3/LAM clinical strains.

**Figure 1:**
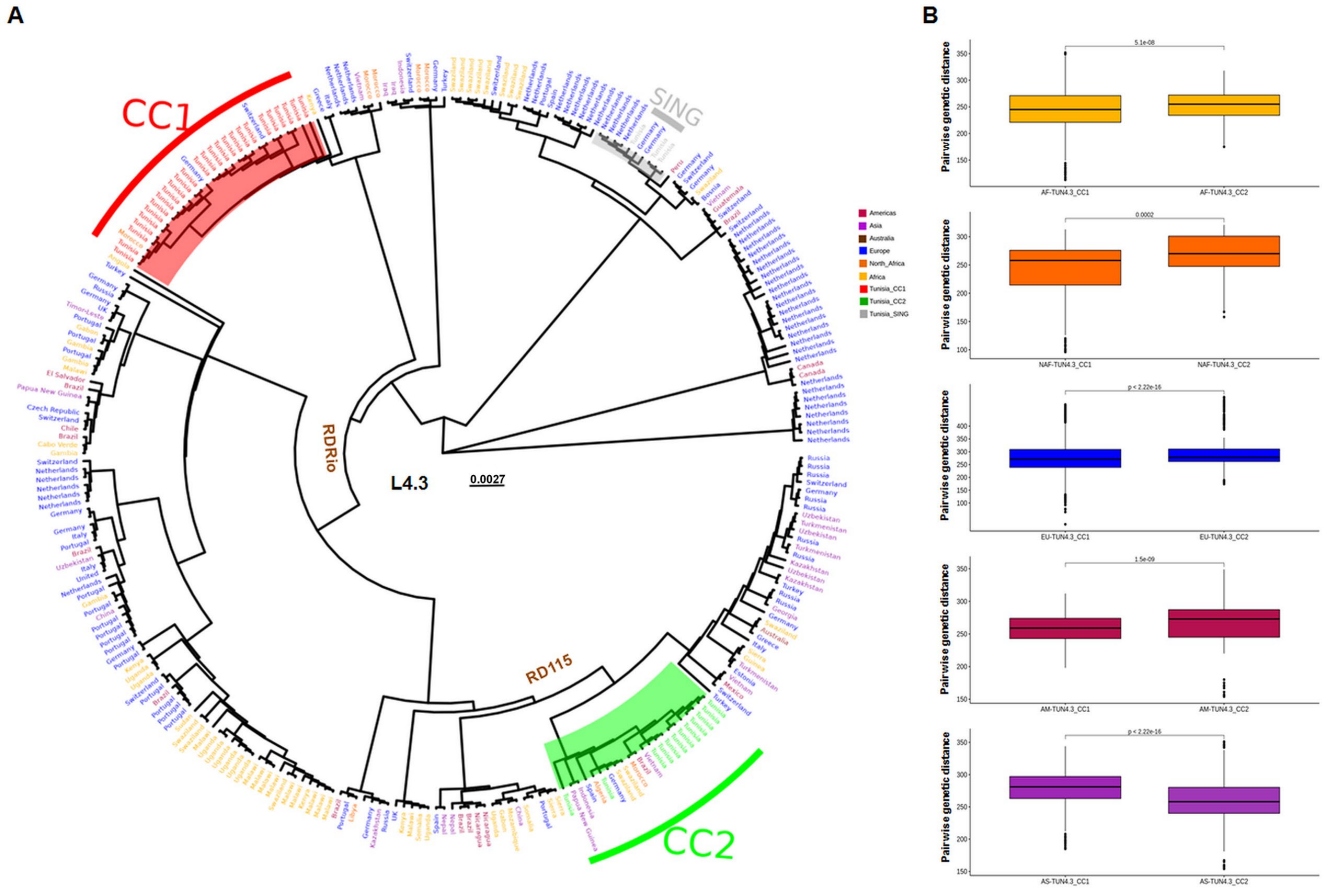
Placement of Tunisian strains within a global L4.3/LAM WGS-based phylogeny. **(A)** Maximum-likelihood phylogeny of 278 L.3/LAM *Mycobacterium tuberculosis* isolates based on 15,575 variable sites. The origin of isolates are indicated and colored according to their continent. Northern Africa (NAF) is separated from the rest of the continent. The ancestral nodes of the RDRio and RD115 deletions are indicated. **(B)** Box plots of pairwise genetic distances (number of polymorphisms) for each TUN4.3_CC. Colors indicate the origin of strains: AF = Africa, NAF = North Africa, EU = Europe, AM = Americas and AS = Asia.

TUN4.3_CC1 and TUN4.3_CC2 proved distantly related to each other, with all strains belonging to each one of these CCs being clustered together (Figure 1A). While the TUN4.3_CC2-containing clade included globally widespread L4.3/LAM strains (Africa, Europe, South America, Asia, Oceania), which all harbored the ancestral RD115 deletion, the one entailing TUN4.3_CC1 proved geographically restricted. Indeed, aside from including all TUN4.3_CC1 isolates, it comprised only one Moroccan and two European strains, with neither RD115-nor RDRio-deleted strains. The genomes of the 3 singletons were very closely related to each other, but clearly diverged from the two main CCs. Pairwise genetic distance comparisons further confirmed the close relatedness of TUN4.3_CC1 to L4.3/LAM strains originating from Africa or Europe (Figure 1B).

### Phylogeographical origin of L4.3/LAM prevailing CCs

**Figure 2:**
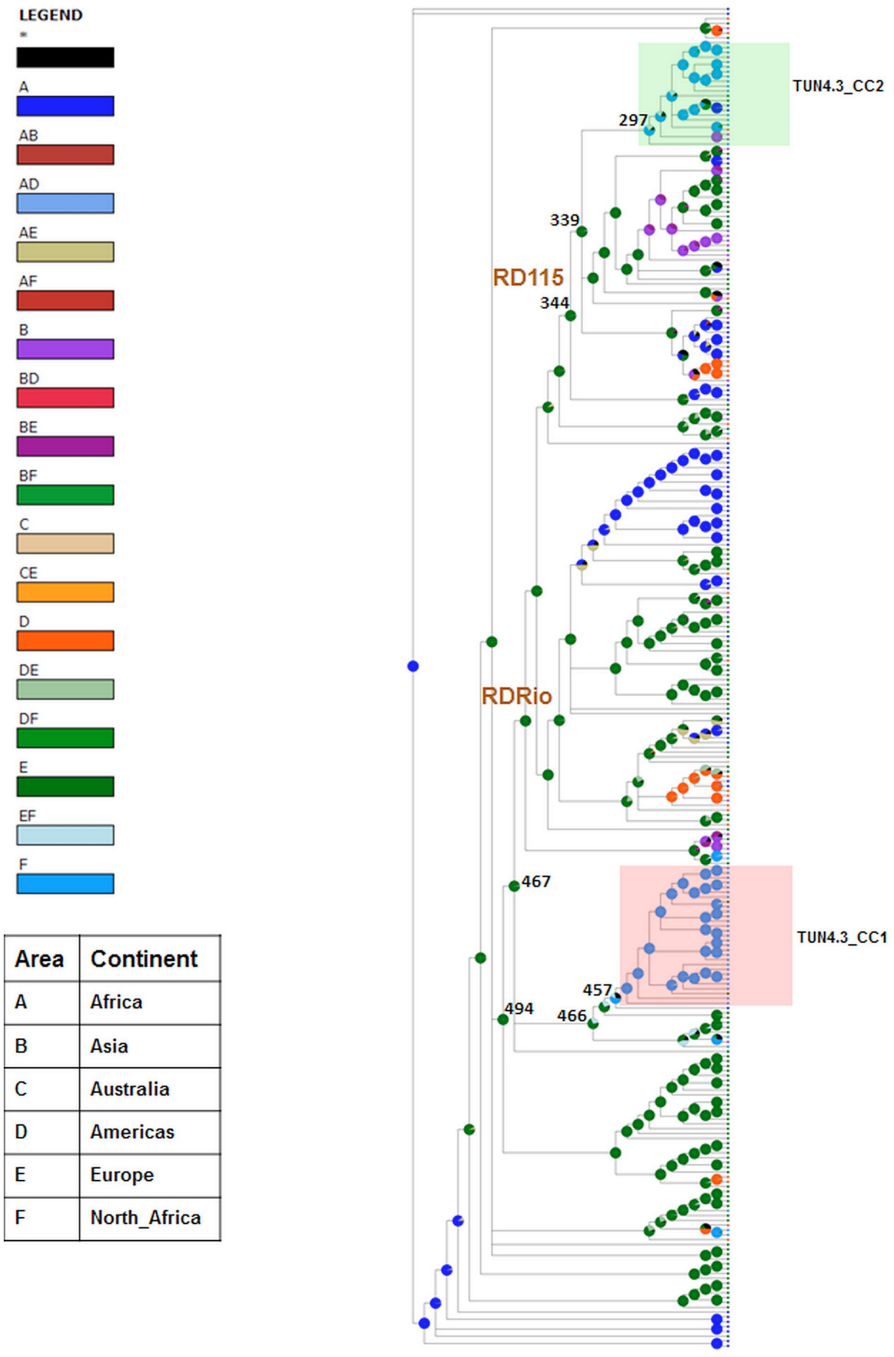
Phylogeographical origin of L4.3/LAM prevailing CCs. Graphical results of ancestral distribution areas at each node of the phylogeny obtained by BBM (RASP). RDRio and RD115 deletions are shown for nodes. Color key to predict possible ancestral ranges: A = Africa, B = Asia, C = Australia, D = America, E = Europe and F = North Africa; letter combinations indicate a combination of ranges; black with an asterisk represents other ancestral ranges. Nodes of interest are indicated by their number.

The geographical origin of L4.3/LAM strains circulating in Tunisia was assessed using BBM and S-DIVA, both of which yielded almost congruent results, albeit with different marginal probabilities. Europe was identified as the most likely origin for TUN4.3_CC2. Indeed, this CC is part of the RD115 clade that descended from node 344 (Figure 2), whose European origin was confirmed by BBM (ancestral area: 97.4%; probability: 0.88) and S-DIVA, yet with a low probability for the latter method (ancestral area: 80.6%; probability: 0.21). With regard to the predominant TUN4.3_CC1, both methods predicted a Euro-North African origin at node 466 (Figure 2), from which the clade containing TUN4.3_CC1 branched off, but with a low probability support (ancestral area: 68.5% Europe and 29.3% Europe/North Africa with a probability of 0.2 for BBM; ancestral area: 25% Europe, 25% North Africa, 25% Europe/North Africa, and 25% Europe/North Africa/Africa with a very low probability of 0.02 for S-DIVA). Strikingly, both BBM and S-DIVA assigned a North African origin for the TUN4.3_CC1 most recent common ancestor (MRCA) (node 457) with a high probability (0.97 and 1.00, respectively). This contrasts with TUN4.3_CC2, whose MRCA (node 297) could have originated either from Europe, North Africa, or Asia.

### Evidence of positive selection

We compared patterns of selective pressure among the different gene categories of TUN4.3_CC1. Strong evidence of positive selection acting on genes encoding pathways of cell wall and cell processes was revealed by LRT for both *site* and *branch-site* models (*p* = 8.423e-12 and 1.9e-21, respectively) (Table 1). The latter model also detected a strong evidence of positive selection in the gene categories “virulence, detoxification, adaptation” and “lipid metabolism (*p* = 0.00027 and 0.000, respectively), while values close to significance resulted from the *site* model for the gene categories “conserved hypothetical proteins” and “virulence, detoxification, adaptation.”

**Table 1:** Likelihood values and parameter estimates from PAML *site* and *branch-site* analyses.

^a^Gene category	Nb of genes	Nb of SNPs	Nb of sSNPs	Nb of nsSNPs	Site model			Branch-site model	
					*p value* M1a vs. M2a M7 vs. M8	^b^PSS	*Site(s) ω Ratio* *± SE*	Foreground TUN4.3_CC1 *ω_2_*	*p value* M2b vs. M2a
Cell wall & cell processes	233	184	65	119	8.423e-12	*ponA1* P631S* esxK A58T^*^ ceoB T117A^**^ sugI P423L^**^ esxV Q20L* esxV S23L^**^ eccC2 D788N*	8.789 + − 1.418	1.777	1.9e^−21^
Conserved hypothetical proteins	326	275	110	165	0.018	–	–	0	1.0
Information pathways	55	35	10	25	0.223	*sigM* S78W*	6.180 + − 2.714	1.699	0.456
Intermediary metabolism & respiration	209	121	43	78	0.135	–	–	0	0.51
Lipid metabolism	95	93	46	47	0.082	–	–	1.805	0.00
Regulatory proteins	184	44	18	26	1.000	–	–	19.65	0.307
Virulence, detoxification, adaptation	221	37	17	20	0.049	*vapC47* S46L^**^(100)	8.412 + − 2.188	3.418	0.00027

a Gene categories were defined as in http://tuberculist.epfl.ch/.

b PSS, Positive Selected Sites with BEB Posterior Probability of *ω* > 1 (^*^p > 95%; ^**^p > 99%).

Consistent with LRT results, BEB analysis under the *site* model further identified nine putative positively selected sites, seven of which map to the gene category “cell wall and cell processes” with a posterior probability >95% (Table 1). It is worth mentioning that four of these putative positively selected sites are encoded by genes of the type VII secretion system (*esxK*, *esxV*, and *eccC2*).

**Figure 3:**
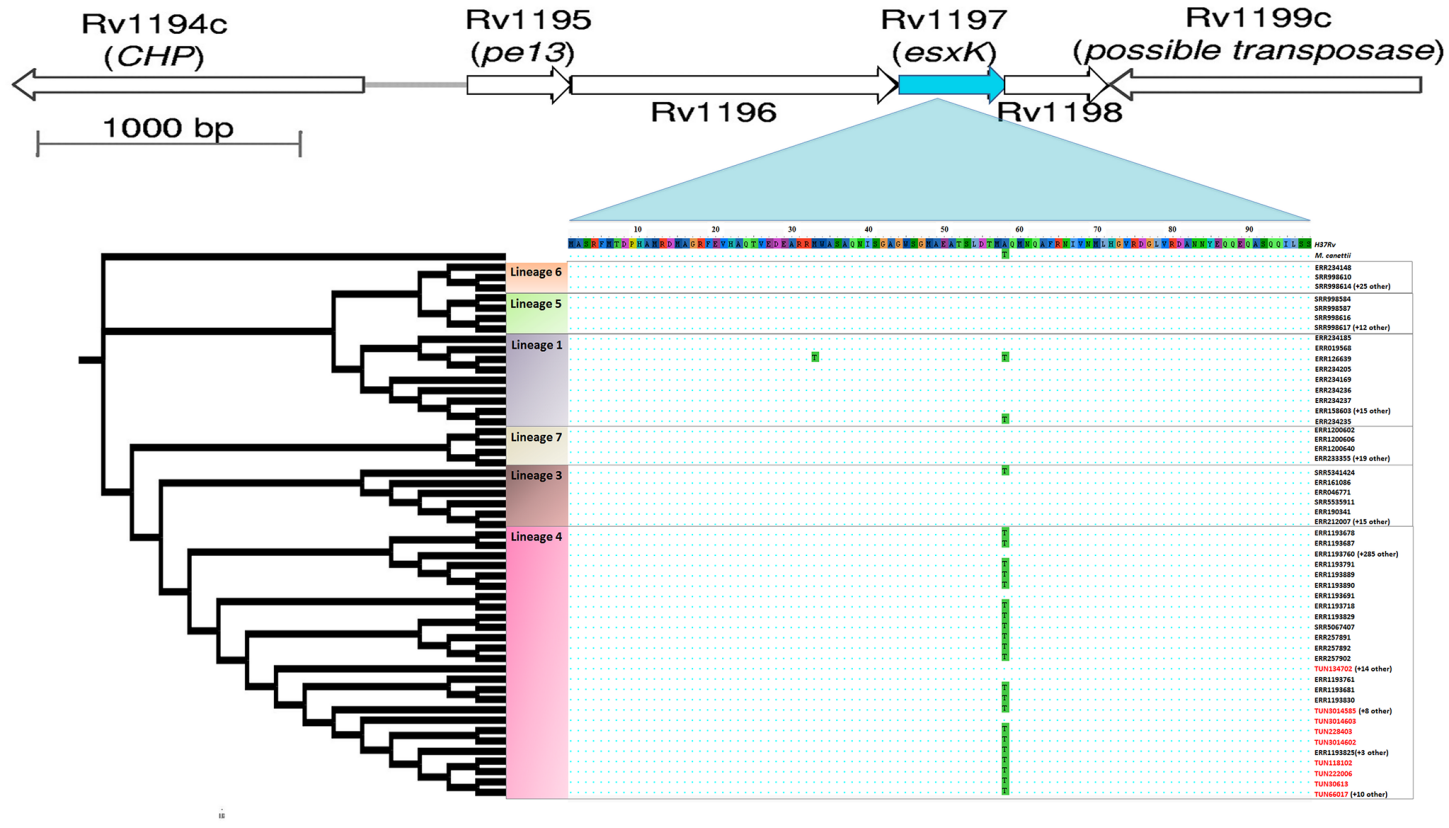
Position of homoplastic A58T *esxK* nsSNP in cladogram subtree of 458 *M. tuberculosis* isolates. The maximum likelihood tree was constructed using IQ-TREE, which included six major lineages and *M. canetti* as outgroup. *esxK* and *esxL* (Rv1198) gene pair is located immediately downstream of pe13 and ppe18 gene pair.

**Figure 4:**
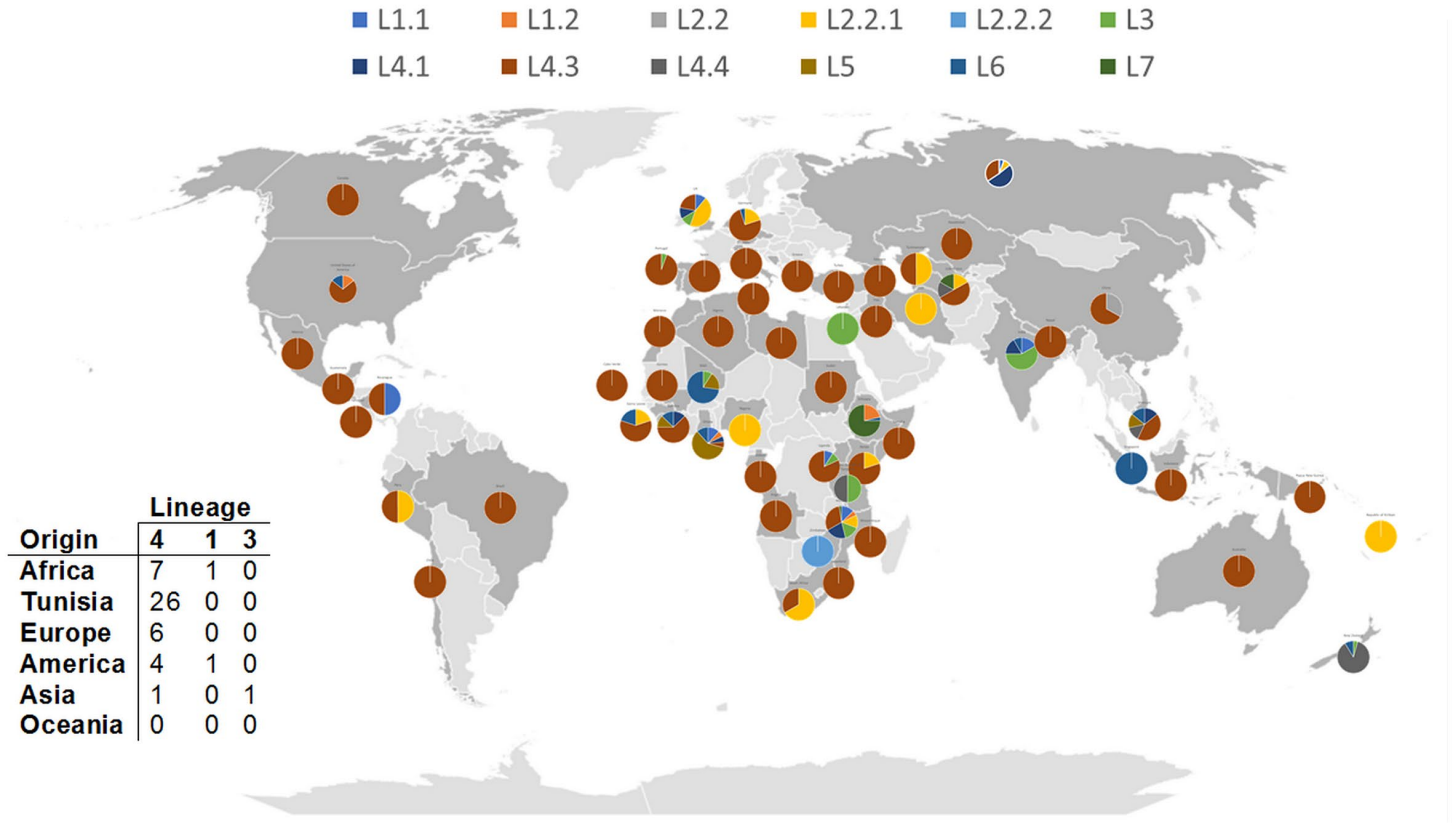
Distribution of A58T *esxK* nsSNP across *M. tuberculosis* lineages from different geographical regions. Each pie chart segment reflects the relative proportion of selected *M. tuberculosis* lineages for each country (see color chart for the respective major lineages). Number and origin of isolates harboring A58T nsSNP in different lineages are illustrated in the table. 0.0027.

Among the nine putative positively selected amino acid changes, three (*esxK* A58T, *eccC2* D788N, and *sigM* S78W) are not shared with TUN4.3_CC2 (Table 1). The *esxK* A58T mutation was found to be common to all TUN4.3_CC1 isolates, and could also be identified frequently in several L4 sublineages and in some isolates of L1 and L3 from various geographic areas worldwide, thus suggesting its homoplasic nature (Table 2; Figures 3, 4). By contrast, *eccC2* D788N appears to be specific to TUN4.3_CC1. Of note, this mutation is close to fixation, being present in 96% of the TUN4.3_CC1 population (Table 2). However, and despite highly significant LRT values, none of these putative positively selected sites could be identified under the *branch-site* model.

**Table 2:** Frequency of positively selected sites (PSS) across *Mycobacterium tuberculosis* sublineages.

PSS	TUN4.3_CC1 Frequency (%)	TUN4.3_CC2 Frequency (%)	Sublineages frequency (%)											
*ponA1* P631S	7.69	100	L1.1	L3	L4.1	L4.3	L4.4							
			7.14	6.45	83.33	53.26	26.09							
*ceoB* T117A	100	100	L1.1	L1.2	L2.2	L2.2.1	L2.2.2	L3	L4.1	L4.3	L4.4	L5	L6	L7
			100	100	100	100	100	61.29	93.75	97.46	95.65	94.12	20.69	30.43
*sugI* P423L	100	100	L1.1	L1.2	L2.2	L2.2.1	L2.2.2	L3	L4.1	L4.3	L4.4	L5	L6	L7
			100	100	100	100	100	67.74	95.83	99.28	91.3	94.12	96.55	100
*eccC2* D788N	96.15	0	L4.3											
			15.58											
*sigM* S78W	23.07	0	L4.3											
			2.54											
*VapC S47L*	100	100	L1.1	L1.2	L2.2.1	L2.2.2	L3	L4.1	L4.3	L4.4	L5	L6	L7	
			100	100	100	100	64.51	95.83	99.27	95.65	100	96.55	100	
*esxK* A58T	100	0	L1.1	L3	L4.3									
			14.28	4.76	10.14									
*esxV* Q20L	100	100	L1.1	L1.2	L2.2	L2.2.1	L2.2.2	L3	L4.1	L4.3	L4.4	L5		
			71.43	50	100	72.41	100	35.48	68.75	69.2	47.83	52.94		
*esxV* S23L	100	100	L1.1	L1.2	L2.2	L2.2.1	L2.2.2	L3	L4.1	L4.3	L4.4	L5		
			71.43	50	100	72.41	100	32.26	66.67	69.93	52.17	64.71		

Hence, we scanned further the genomes for homoplastic mutations, which is the most indicative of positive selection in *M. tuberculosis*, given its highly clonal nature. Strikingly, the *site* model-derived *esxK* A58T mutation was identified, thus confirming its homoplastic nature. We also noticed the frequent occurrence of the Rv0197 nonsense mutation, 234477TG, which has previously been shown to be associated with enhanced transmissibility *in vivo* (Nebenzahl-Guimaraes et al., 2017). This mutation occurred repeatedly 9 times in TUN4.3_CC1 (53.8%), 4 times in TUN4.3_CC2 (25%), and 2 times among singletons (data not shown).

Next, given the fact that *esx* genes seem to be particularly subject to selective pressure, we scrutinized the whole 23-membered gene family in detail and identified several nsSNPs (Table 3). Strikingly, the two positively selected changes in *esxV*, Q20L and S23L, which have already been reported (Uplekar et al., 2011), affect known epitopes, with Q20L being also present in an epitope of the *esxL*. These mutations could be identified through *M. tuberculosis* L1 to L5. Curiously, *esxV* Q20L and *esxV* S23L mutations are distributed almost evenly through L1 to L5, with a frequency varying from 100% (L2) to 32% (L3) (Table 2). In Tunisia, *esxV* Q20L and *esxV* S23L are present in all isolates of both TUN4.3_CC1 and TUN4.3_CC2 (Table 1).

**Table 3:** SNPs occurring in *esx* genes epitopes.

Gene	SNP	AA change	Overall number of isolates	TUN4.3_CC1 (*n*)	TUN4.3_CC2 (*n*)	Singletons (*n*)
Rv0288|esxH	C276T	A92A	2	2	0	0
Rv1197|esxK	T126C	G42G	21	21	0	0
Rv1197|esxK	G172A	A58T	26	26	0	0
Rv1197|esxK	G258A	E86E	1	0	1	0
Rv1198|esxL	C39T	H13H	4	0	1	3
Rv1198|esxL	**A59T**	**Q20L**	**3**	**0**	**0**	**3**
Rv1198|esxL	A94G	I32V	1	0	1	0
Rv1198|esxL	A109G	T37A	1	0	1	0
Rv1198|esxL	A115G	S39G	1	1	0	0
Rv1198|esxL	C143T	A48V	1	0	1	0
Rv3619c|esxV	C169T	L57L	40	26	12	2
Rv3619c|esxV	**C68T**	**S23L**	**41**	**26**	**12**	**3**
Rv3619c|esxV	**A59T**	**Q20L**	**41**	**26**	**12**	**3**
Rv3619c|esxV	C39T	H13H	37	23	12	2
Rv3620c|esxW	A258G	E86E	37	23	11	3
Rv3620c|esxW	A4G	T2A	16	6	10	0
Rv3891c|esxD	G274A	D92N	1	0	1	0
Rv3905c|esxF	G311A	#104#	1	1	0	0

SNPs affecting known epitopes are in bold.

Moreover, by applying the SIFT algorithm, we identified numerous mutations in both CCs that might affect protein function. In this study, we subjected to SIFT only those mutations that occurred in coding sequences and which tended to be fixed in the strain population (observed in ≥80% of isolates). A complete list of the deleterious mutations identified in the two main CCs (13 and 12 nsSNPs in TUN4.3_CC1 and TUN4.3_CC2, respectively) is provided in supplementary data. For both CCs, it is worth mentioning that the gene categories “conserved hypotheticals” and “intermediary metabolism and respiration” accounted for a large number of functionally impactful mutations (76.92 and 66.66% for TUN4.3_CC1 and TUN4.3_CC2, respectively).

## Discussion

The current global picture of the MTBC epidemiology reflects a strong association with the human host. However, it is obvious that TbD1-deleted “modern” lineages, L2 and L4, are more successful in terms of geographical spread and epidemic potential compared with TbD1-intact “ancestral” lineages (Gagneux, 2012; Comas et al., 2013; Stucki et al., 2016; Bottai et al., 2020). Detailed population genetics studies further uncovered the relative success of particular MTBC clones or strains in specific host populations. Such a relative success is likely to be due to a higher transmission fitness and/or the consequence of local adaptive evolution as evidenced by the identification of positively selected mutations in the *M. tuberculosis* genome, and the observed interactions between human genetic polymorphisms and *M. tuberculosis* genotypes (Comas et al., 2013; Brites and Gagneux, 2015; Merker et al., 2015; Brynildsrud et al., 2018; Holt et al., 2018; Chiner-Oms et al., 2019; Menardo et al., 2021). Hence, focusing on *M. tuberculosis* clones that successfully expand in particular geographic regions may help better delineating the underlying molecular basis. Here, by performing population genetics analyses of the most predominant L4.3/LAM clonal complex in Tunisia, TUN4.3_CC1, we point to key genomic changes that could have contributed to its relative success, highlighting the prominent involvement of genes of the ESX/Type VII secretion system.

Overall, phylogenetic analyses established an obvious link of the current Tunisian L4.3/LAM population and strains originating from Europe, thus lending further support to the prevailing scenario according to which the L4.3/LAM sublineage originated in Europe and subsequently spread to Africa and the Americas through European colonial migrations (Mokrousov et al., 2005; Stucki et al., 2016; Brynildsrud et al., 2018; O’Neill et al., 2019).

However, previous studies have invoked a Maghrebian origin for the L4.3/LAM sublineage (Mokrousov et al., 2005). Given the very likely African origin of L4 (O’Neill et al., 2019), one should not dismiss such a hypothesis. Indeed, a subset of L4.3/LAM strains could have originated locally or imported to the Maghreb through regional land routes linking Eastern, Western, and Northern Africa, particularly during the era of Roman conquest (O’Neill et al., 2019). Such a possibility turns out to be very plausible, since our data collectively converge toward a local origin of TUN4.3_CC1, the most prevalent L4.3/LAM clonal complex in Tunisia and, most likely, in the Maghreb, as well. Indeed, TUN4.3_CC1 displayed reduced levels of genetic diversity, a hallmark of small populations with restricted geographical ranges (Ellstrand and Elam, 1993). The absence of the most ancient deletion, RD115, indicated its closest relationship with the L4.3/LAM progenitor. In addition, the finding that TUN4.3_CC1 was genetically closest to the pool of L4.3/LAM strains from Africa and Europe, argue for its early history. Moreover, our phylogeographical analysis robustly pointed to a North African origin of the MRCA of TUN4.3_CC1, lending further support that it has evolved locally. This contrasts with the majority of other geographical locations where rapidly expanding L4.3/LAM genotypes, notably the RDRio genotype, are of recent origin, being invariably linked to European colonial migrations (Brynildsrud et al., 2018).

Aside from having evolved locally, TUN4.3_CC1 has been undergoing a demographic expansion for almost 131 years (Skhairia et al., 2023). Such a long diversification time could have resulted in the accumulation and/or fixation of favorable mutations, making it the most predominant clonal complex of the L4.3/LAM sublineage in Tunisia. Additionally, the fact that TUN4.3_CC1 was not particularly associated with drug resistance, a factor that might be compensated by an increased fitness, argues toward its intrinsic ability to spread successfully in the host population (Skhairia et al., 2021). This has prompted us to carry out a genomewide scan for positive selection in TUN4.3_CC1. Our data unambiguously detected strong signals of positive selection acting on the gene category “cell wall and cell processes” as witnessed by the highly significant LRT of models comparisons under both *site* and *branch-site* models. Yet, none of the positively selected sites identified under the *site* model could be confirmed by BEB analysis when TUN4.3_CC1 was considered as the foreground branch and the remaining L4.3/LAM strains as the background in a *branch-site* test. Actually, in view of the distribution and frequency of the putative positively selected sites across L4.3/LAM sublineages and other lineages (Table 2), one can rightfully argue that the *site* model-derived positively selected residues are rather of phylogenetic significance, inasmuch as *M. tuberculosis* is a highly clonal pathogen. Indeed, with the exception of the mutation in *eccC2*, all the other mutations occurred independently in different lineages, some of which occur in almost the entire strain collection (*ceoB* T117A, *sugI* P423L, *esxV* Q20L, *esxV* S23L). However, among the positively selected sites identified under the *site* model, at least two mutations map to genes, or gene homologs, that have previously been shown to be subject of positive selection (*PonA1*, *esxK*, and *esxV*).

The A58T mutation in *esxK*, an *esx* gene of the ESX-5 locus, is of particular interest since it occurred almost exclusively in the prevalent TUN4.3_CC1, being shared by all isolates of this clonal complex. The most likely scenario is that *esxK* A58T occurred in the ancestor of this clonal complex, and then underwent a clonal spread. Because this mutation was found to be homoplasic, one can rightfully argue that it may have potentially contributed to TUN4.3_CC1 successful expansion and/or local adaptation.

Another mutation that might account in the relative success of TUN4.3_CC1 is the one that took place in *eccC2* gene (*eccC2* D788N), which encodes an ATPase that is essential to the functioning of the ESX-2 secretion locus. Importantly, this amino acid replacement likely evolves toward fixation in TUN4.3_CC1, but it could be identified in none of TUN4.3_CC2 isolates. Hence, though *eccC2* D788N did not prove homoplasic, one should not dismiss its potential contribution in the expansion of TUN4.3_CC1. In the same vein, the *sigM* S78W mutation, which affects a gene known to positively regulate the expression of *esx* genes, might be of significance in the evolutionary success of TUN4.3_CC1 relative to TUN4.3_CC2.

Taken together, our findings lend further support to the mounting evidence that inheritance or *de novo* acquisition of beneficial mutations in the ESX/Type VII secretion system is prominently involved in the enhanced transmission fitness and/or adaptation of *M. tuberculosis* (Ates et al., 2016; Holt et al., 2018; Meehan et al., 2019; Vaziri and Brosch, 2019). Indeed, detection of positive selection signals in genes of the ESX/Type VII secretion system of successful and/or locally adapted *M. tuberculosis* genotypes is not without precedent. A mutation in *esxW*, an *esx* member of the ESX-5 locus that is paralogous to *esxK*, has previously been deemed to be involved in the enhanced transmission of Beijing lineage of *M. tuberculosis* in Vietnamese populations (Holt et al., 2018). On the other hand, signatures of positive selection at *esxH*, a gene belonging to the ESX-3 locus, has been correlated with adaptation of *M. tuberculosis* L1 and L3 lineages to the Indian Ocean rim (Menardo et al., 2021). Such a finding is in line with the critical role of EsxH protein in inhibiting the ability of macrophages and dendritic cells to activate Mtb antigen-specific CD4+ T cells (Portal-Celhay et al., 2016). Here, we describe a new homoplasic mutation in *esxK,* a member of the ESX-5 secretion locus, which has accompanied the evolution of a successful clonal complex. The fact that *esx* genes of different loci were found to function in concert (Pajuelo et al., 2021), raises the possibility that the identified mutations in *esxK* and *eccC2* of the successful TUN4.3_CC1 could, in fact, be interrelated.

Furthermore, other favorable mutations could have contributed to the successful expansion of TUN4.3_CC1. Several novel mutations in TUN4.3_CC1 predicted to be highly impactful (SIFT score: 0.000–0.005) (Supplementary Table S2) were found to be associated with genes directly involved in *M. tuberculosis* virulence/adaptation (*eccD1*, *eccE5*, *ctpF*, *aceA*, *mce3*, *ephB*), or known to be critical for adaptation to new ecological niches, such as methionine biosynthesis (*metH*), fatty acid catabolism (*fadE21* and *fadD31*), and DNA repair and replication (*recD* and *dnaG*) (Bretl et al., 2011; Cambier et al., 2014). Moreover, the frequent and independent occurrence in TUN4.3_CC1 of the *in vivo* enhanced transmission-associated mutation in Rv0197 (234477TG), could have contributed to its evolutionary success. Of note, Rv0197 is a possible oxidoreductase, classified in the gene category “Intermediary metabolism & respiration.” In this regard, it is worth mentioning that local adaptation of L4 strains has previously been attributed to mutations in genes involved in respiration, such as the *lldD2* promoter mutations, which have been shown to confer a significant benefit in terms of transmissibility (Brynildsrud et al., 2018). Since H37Rv was only used as a reference genome in this study, virulence loci absent in H37Rv could be missed (Gautam et al., 2017).

## Conclusion

To sum up, this study explored the origin and the evolutionary history in Tunisia of L4.3/LAM, the most globally widespread L4 sublineage of *M. tuberculosis*, and the most successful in the Maghreb region. We found that the success of this sublineage in Tunisia stems essentially from a single, locally evolved clone, TUN4.3_CC1, whose branch showed strong evidence of positive selection, particularly acting on the gene category “cell wall and cell processes.” Several mutations in this gene category could have contributed to the success of TUN4.3_CC1, notably those in genes of the Type VII secretion system. Identification of such an ecological specialist will certainly improve our knowledge on the process leading to the emergence of highly fit *M. tuberculosis* clones.

## Data availability statement

The whole genome sequence of the 31 L4.3/LAM Tunisian isolates has been deposited in GenBank under project number PRJEB39509.

## Ethics statement

No interventions were performed for the specific need of this study. Only previously published, fully anonymized WGS data are used in the present study, and hence no further ethical clearance was required.

## Author contributions

HM conceived and designed this study. ND performed genomics, phylodynamics, and statistical analyses. ND and HM analyzed and interpreted the data and wrote the manuscript. All authors contributed to the article and approved the submitted version.

## Funding

This study received financial support from the Tunisian Ministry of Higher Education and Scientific Research under grant LR16IPT01, and benefited of the capacity-building program implemented in the context of PHIND*access*, a European Commission H2020-funded project (project ID: 811034).

## Conflict of interest

The authors declare that the research was conducted in the absence of any commercial or financial relationships that could be construed as a potential conflict of interest.

## Publisher’s note

All claims expressed in this article are solely those of the authors and do not necessarily represent those of their affiliated organizations, or those of the publisher, the editors and the reviewers. Any product that may be evaluated in this article, or claim that may be made by its manufacturer, is not guaranteed or endorsed by the publisher.

## Supplementary material

The Supplementary material for this article can be found online at: https://www.frontiersin.org/articles/10.3389/fmicb.2023.1159994/full#supplementary-material

## Footnotes

^1^ http://www.novocraft.com

## Abbreviations

TB: Tuberculosis
LAM: Latin American & Mediterranean
MIRU-VNTR: mycobacterial interspersed repetitive units-variable numbers of tandem repeats
CC: clonal complex
MST: minimum spanning tree
WGS: whole genome sequence
SNP: single nucleotide polymorphism
sSNP: synonymous single nucleotide polymorphism
nsSNP: nonsynonymous single nucleotide polymorphism
dN/dS: ratio of nonsynonymous changes to synonymous changes
PAML: Phylogenetic Analysis by Maximum Likelihood
