# Supplementary figures and images for: Genomic changes underpinning the emergence of a successful Mycobacterium tuberculosis Latin American and Mediterranean clonal complex
Source: Front Microbiol. 2023 Jun 22;14:1159994. doi: 10.3389/fmicb.2023.1159994 (PMC10325029; doi:10.3389/fmicb.2023.1159994)

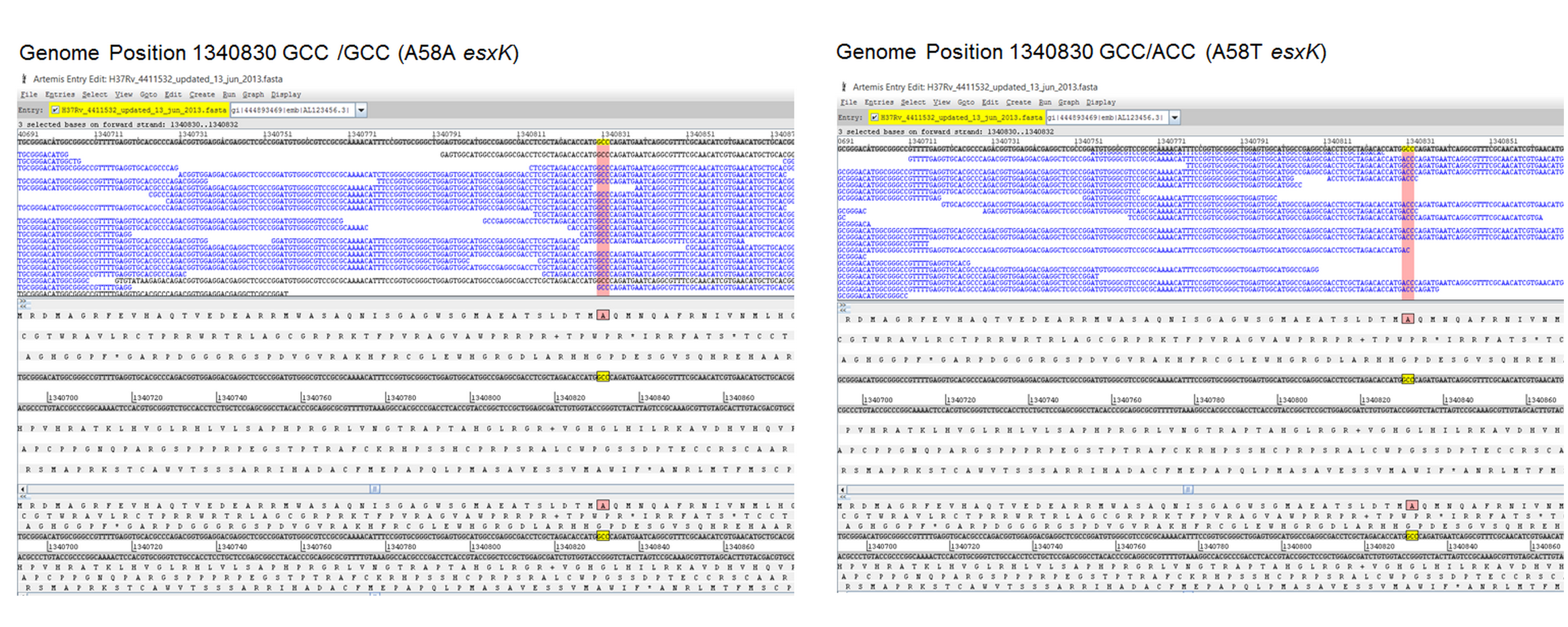

Supplement: Supplementary file 2 [file Image_1.tif]
